# Ureterovaginal fistula: a case report and review of an unexpected contemporary complication secondary to transvaginal ultrasound-guided oocyte retrieval

**DOI:** 10.5935/1518-0557.20230063

**Published:** 2024

**Authors:** Gabriel Chahade Sibanto Simões, Dalmo de Barros e Silva, Joao Roberto Paladino Júnior, Guilherme Lamacchia, Alexandre Gomes Sibanto Simões

**Affiliations:** 1 Urology Department, University of Campinas - UNICAMP, Campinas, SP, Brazil; 2 Urology Department, Faculdade de Ciências Médicas da Santa Casa de São Paulo, São Paulo, SP, Brazil; 3 Urology Department, Centro Universitário FMABC, Santo André, SP, Brazil; 4 Urology Department, Hospital Ipiranga, São Paulo, SP, Brazil

**Keywords:** transvaginal oocyte retrieval, ureteral injury, ureteral trauma, ureterovaginal fistula, *in-vitro* fertilization

## Abstract

Despite the widespread use of transvaginal ultrasound-guided oocyte retrieval in assisted reproductive technology procedures, there is a lack of systematic data on the incidence and nature of its complications. This makes it difficult for healthcare providers to fully understand and manage the risks associated with the procedure, and for patients to make informed decisions about their care. Ureteral injuries and other complications during oocyte retrieval are important to consider and manage appropriately. Early ureterovaginal fistula is a rare but serious complication that can occur after oocyte collection by transvaginal ultrasound. It is important for medical professionals to be aware of this potential complication and to take appropriate measures to prevent and manage it. Minimally invasive treatments for ureterovaginal fistula can be effective in resolving the condition and minimize the risk of further complications. However, early diagnosis and prompt intervention are critical in achieving a successful outcome.

## INTRODUCTION

Although contemporary series show a very low incidence of complications related to oocyte puncture guided by transvaginal ultrasound, we cannot consider it to be a risk-free procedure, and it should not be underestimated because some complications, even though rare, can be serious and even fatal (Levi-Setti *et al*., 2018). Fortunately, most of the reported complications for this procedure are minor, such as self-limited vaginal bleeding and pelvic infection (Ludwig *et al*., 2006).

However, a small number of case reports and reviews of published data have identified injuries to adjacent organs and vessels after oocyte collection (Bennett *et al*., 1993). Therefore, despite its simplicity and effectiveness, patients should be counseled about the possible risks of oocyte retrieval. With the patient’s consent to having her case published, we highlight an early ureterovaginal fistula three days after the oocyte retrieval procedure.

## CASE REPORT

**Figure 1:**
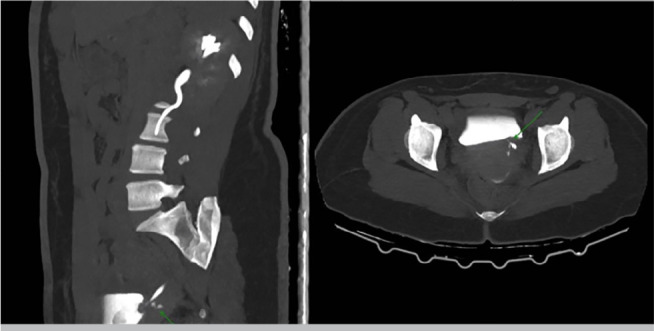
Computed tomography with intravenous contrast demonstrates a fistula extending from the distal left ureter to the vagina.

**Figure 2:**
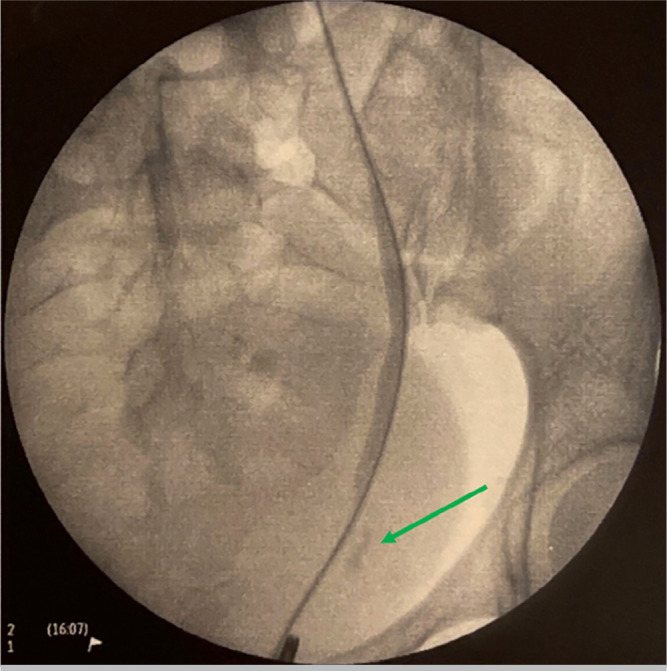
Retrograde pyelogram with extravasation of contrast from the left distal ureter.

A 35-year-old woman, two days after oocyte collection by transvaginal ultrasound, presented to the emergency department with involuntary, continuous, and painless urinary loss. She was diagnosed with infertility with no apparent cause and was in her first *in vitro* fertilization cycle (IVF). The patient did not present a significant past medical history of abnormal Papanicolau smears, abdominopelvic surgeries, pelvic infections, or endometriosis. During the physical examination, the speculum showed urine output in the vaginal dome, and it was not possible to clearly identify the fistulous orifice. Infusion of methylene blue into the bladder by urinary catheter did not show vaginal extravasation, and the method did not identify a vesicovaginal fistula. Computed tomography showed diffuse dilation of the left ureter with extravasation of contrast at the level of the distal third of the vaginal vault, suggestive of ureterovaginal fistula (Figure 1). Cystoscopy examination did not identify bladder lesions or extravasation during the diagnosis procedure. Subsequently, an ascending pyelography was performed on the left, identifying a ureterovaginal fistula due to inadvertent injury to the distal ureter (Figure 2).

Afterwards, the patient underwent an uncomplicated left ureteral stent placement, which was then removed within three weeks, with complete closure of the fistula on follow-up scans.

## DISCUSSION

Currently, ultrasound-guided transvaginal access is the most common approach used to collect oocytes during IVF cycles. This technique was first performed in 1983 (Gleicher *et al*., 1983) and has gained widespread popularity as it is relatively easy to learn and much less invasive compared to the laparoscopic or transabdominal routes, making it now the gold standard for oocyte collection. While it is generally considered safe, as with any medical procedure, there are potential complications that can occur.

Due to the training and standardization of the procedure, complications after follicular aspiration guided by transvaginal ultrasound are currently rare, making it difficult to assess their true incidence. Although the incidence of problems with the technique is relatively small, the attending physician must be aware that they exist and many times they can be serious or even unfortunately fatal. Some of the possible complications of transvaginal ultrasound-guided oocyte retrieval include vaginal bleeding, infection, ovarian hyperstimulation syndrome (OHSS), and damage to surrounding structures. Other complications described include adnexal torsion, rupture of endometriotic cysts, anesthetic complications, and even vertebral osteomyelitis. Over the past two decades, several reports have described the complications associated with this technique and have attempted to address risk factors and safety concerns (El-Shawarby *et al*., 2004).

Ureterovaginal fistula (UVF) is a rare but possible complication that can occur after transvaginal ultrasound-guided oocyte retrieval (Coroleu *et al*., 1997; Mongiu *et al*., 2009; Spencer *et al*., 2017; von Eye Corleta *et al*., 2008). While unusual, it is possible for the probe to accidentally puncture the vaginal wall and cause damage to nearby organs, such as the ureter.

It is important to note that the ureter is located close to the anterior fornix of the vagina and can potentially be injured during transvaginal oocyte retrieval (TVOR). However, ureteral injury during TVOR is relatively uncommon, despite the fact that the ureter passes over the anterior fornix of the vagina.

Anatomic distortion caused by conditions such as endometriosis, pelvic inflammatory disease, prior gynecologic surgery, and pressure from the transvaginal ultrasound probe may increase the risk of ureteral injury during TVOR (Grynberg *et al*., 2011). Healthcare providers need to be aware of the potential risks associated with TVOR and take appropriate precautions to minimize the risk of ureteral injury. The present case had no previous pelvic or abdominal surgical procedures, and previous imaging studies showed a normal pelvic anatomy.

The presentation of this case is unique in the literature because, unlike previously reported cases of UVF formation after TVOR, in which initial symptoms included severe flank pain, lower abdominal discomfort, vaginal bleeding, and leakage, our patient had an early and painless urinary leakage in the forty-eight hours after the procedure, with no other signs or symptoms in the anamnesis and physical examination.

Treatment for ureterovaginal fistula typically involves surgery to repair the damaged tissue and restore normal function to the affected organs, and antibiotic prophylaxis may also be prescribed. The injury was successfully treated conservatively with ureteral stent implantation, demonstrating that this treatment option should be considered before more invasive interventions such as nephrostomy tube placement or open surgical repair (von Eye Corleta *et al*., 2008; Spencer *et al*., 2017).

It is important to note that although ureterovaginal fistula is a rare complication, it is still a potential risk associated with transvaginal ultrasound-guided oocyte retrieval and physicians should discuss and expose patients to the potential risks and benefits of treatment and advise them to report any unusual symptoms or discomfort after the procedure.
